# Clinical characteristics and short-term outcomes of left ventricular non-compaction cardiomyopathy in neonates

**DOI:** 10.3389/fped.2026.1774991

**Published:** 2026-03-16

**Authors:** Yan Chen, Hua Peng, Yue Song, Ziang Liu, Yalan Liu

**Affiliations:** 1 Department of Pediatric, Union Hospital, Tongji Medical College, Huazhong University of Science and Technology, Wuhan, China; 2 Department of Ultrasound Medicine, Union Hospital, Tongji Medical College, Huazhong University of Science and Technology, Wuhan, China

**Keywords:** clinical manifestations, left ventricular non-compaction, neonate, outcomes, reversibility

## Abstract

**Background:**

Left ventricular non-compaction (LVNC) is a rare type of cardiomyopathy. It is more difficult to diagnose during the neonatal period. This study reported the clinical manifestations of neonatal LVNC at initial diagnosis and investigated their short-term outcomes.

**Methods:**

A cohort of 10 neonates was enrolled. Their clinical characteristics were analyzed, and follow-up was conducted for 1 year.

**Results:**

The neonates had an average gestational age of 35.82 weeks and an average birth weight of 2,636 g. The average age at initial diagnosis was 12 days and clinical manifestations were highly variable, ranging from asymptomatic (20%) to cyanosis (50%), dyspnea (50%), arrhythmia (60%), and heart failure (30%). Plasma myocardial injury markers were elevated, and electrocardiogram abnormalities were present in 90% of infants. Echocardiography revealed an average left ventricular (LV) non-compacted/compacted ratio of 2.96 and reduced systolic function (ejection fraction: 38.5%; fractional shortening: 18.8%). The average LV internal diameters were both above the normative reference intervals, with an end-diastolic diameter of 2.26 cm and an end-systolic diameter of 1.84 cm. During follow-up, two neonates died. Among the eight survivors, three with non-isolated LVNC underwent cardiac surgery, whereas five were asymptomatic with normal LV systolic function at 1 year of age.

**Conclusions:**

The clinical presentation of neonatal LVNC varies widely, from asymptomatic to those with dyspnea, cyanosis, arrhythmia, or heart failure. In our cohort, heart failure at initial diagnosis was associated with a poorer prognosis. Short-term follow-up suggested that with aggressive management, myocardial function may show signs of reversibility in a subset of neonates.

## 1 Introduction

Left ventricular non-compaction cardiomyopathy (LVNC), also referred to as non-compaction cardiomyopathy, is a cardiomyopathy thought to result from arrest of normal embryogenesis of the endocardium and myocardium (1). It is prominently characterized by abnormal trabecular architecture, with a disproportionately thick layer of trabeculations on the inner aspect of the ventricular wall (2). In mammals, trabeculae undergo a necessary compaction step during fetal development to form a competent myocardial wall (3). Adverse conditions during fetal development can result in permanent changes in the trabeculae. Preterm birth leads to an early switch from fetal to postnatal circulation before completion of left ventricular *in utero* development (4). Meanwhile, cardiovascular remodeling and dysfunction are well documented in fetal growth restriction (5). Thus, interrupted *in utero* maturation in preterm and adaptive remodeling to chronic intrauterine stress in small for gestational age can result in unique hypertrabecular phenotypes. These physiological patterns must be distinguished from pathological LVNC in neonates.

LVNC is widely recognized as an unclassified cardiomyopathy that typically affects the left ventricle (LV). The prevalence of LVNC is 0.05% in adults (6), 0.14% in children (7), and 0.076% in neonates (8) based on echocardiography.

Clinical manifestations of LVNC are highly variable, ranging from asymptomatic cases to disabling congestive heart failure, arrhythmia, systemic thromboembolism, and sudden cardiac death (2). At present, data regarding neonatal LVNC are sparse. Therefore, it makes the diagnosis of LVNC more difficult during the neonatal period. This study describes the clinical characteristics and short-term outcomes of neonates with LVNC. The findings indicated that with aggressive treatment, a subset of these neonates may show potential for reversibility during infancy.

## 2 Materials and methods

### 2.1 Study design and population

This study enrolled infants who were admitted to the neonatal intensive care unit of Union Hospital, Tongji Medical College, Huazhong University of Science and Technology, Wuhan, China, between January 2010 and January 2020. Medical records were reviewed to identify cases of LVNC based on Jenni's criteria (9). Jenni's criteria included (1) numerous, excessively prominent trabeculations and deep intertrabecular recesses; (2) intertrabecular spaces filled by direct blood flow from the ventricular cavity, as visualized on color Doppler imaging; and (3) presence of a two-layer ventricular wall with a non-compacted-to-compacted ratio (NC/C ratio) >2 at the site of maximal wall thickness in end-systolic short-axis view in ≥1 LV segment. Infants who did not have coexisting cardiac abnormalities were diagnosed with isolated LVNC (i-LVNC), while infants with structural heart disease were classified as non-isolated LVNC (ni-LVNC) (9).

### 2.2 Echocardiography

**Figure 1:**
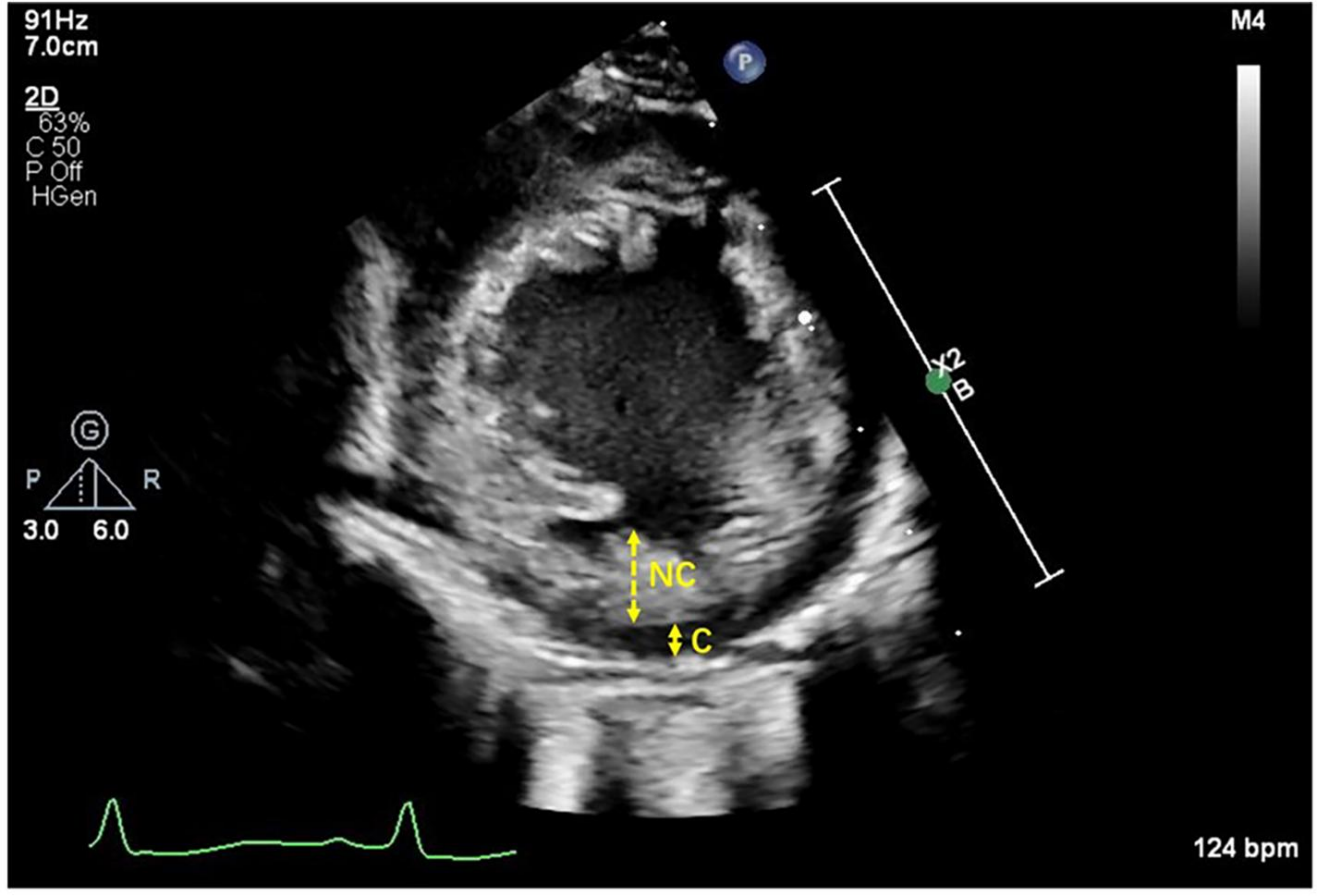
The NC/C ratio was measured in the parasternal short-axis view during end-systole phase. A left ventricular myocardium with two distinct layers, namely, compacted (C) and noncompacted (NC).

Echocardiograms were obtained under the following conditions: (1) development of clinical signs suggestive of cardiac involvement (e.g., respiratory changes, cardiovascular symptoms); (2) routine postnatal evaluation following prenatal suspicion of heart disease; or (3) high-risk screening. Two echocardiographers with ≥5 years of experience in pediatric echocardiography performed all scans. Echocardiographic examinations were conducted using a Philips EPIC 7C system equipped with an S8-3 phased-array transducer (Philips Medical Systems, Bothell, WA, USA). Two-dimensional (2D), Doppler, and M-mode echocardiographic assessments were performed in accordance with standard protocols. Comprehensive echocardiographic views were acquired, including parasternal long-axis, parasternal short-axis, apical two-chamber, apical three-chamber, and apical four-chamber perspectives. The NC/C ratio was measured in the parasternal short-axis view during the end-systole phase, with the non-compacted (NC) and compacted (C) layer thickness measured perpendicular to the compacted layer, based on Jenni's criteria (9) (Figure 1).

### 2.3 Data collection

We collected and analyzed relevant data on clinical presentation and symptoms, details of the clinical course, personal and family histories, myocardial injury markers, 12-lead electrocardiograms (ECGs), and echocardiographic findings. Follow-up of the patients was conducted at our hospital for up to 1 year. The diagnosis of heart failure was based on clinical symptoms of feeding difficulty, tachypnea, and cyanosis, and decreased left ventricular ejection fraction (LVEF) on echocardiography (10). Shock was defined based on the criteria of Baske K (11).

## 3 Results

### 3.1 Patient demographics

This study included 10 neonates diagnosed with LVNC, categorized as isolated (*n* = 6) and non-isolated (*n* = 4) types. Among them, there were six boys and four girls. The cohort had an average gestational age (GA) of 35.82 weeks and an average birth weight of 2,636 g. Of these infants, seven were term and three were preterm; by weight for GA, two were small for gestational age (SGA) and eight were appropriate for gestational age (Table 1). Two of these cases (Case 9 and Case 10) were singletons from twin pregnancies. Two preterm infants experienced neonatal asphyxia at birth. The mothers were healthy, except that the mother in Case 9 suffered from systemic lupus erythematosus (SLE) and hypertension, while the mother in Case 10 had preeclampsia. Family history revealed that the brother of Case 1 died of congenital heart defect (CHD) and the twin sister of Case 10 had a patent ductus arteriosus (PDA), which resolved at 3 months. No other family history of cardiac disorders was reported. Agenesis of the corpus callosum was identified in Case 6 by magnetic resonance (MR) imaging.

**Table 1:** Demographic and clinical characteristics of the patients enrolled in the study.

Patient	1	2	3	4	5	6	7	8	9	10
GA (w)	40	38	33	39 + 2	38	38	40 + 1	31 + 2	34	30
Birth weight (g)	3,000	3,100	2,375	3,900	3,400	2,040	3,660	1,950	1,540	1,400
Sex	M	M	M	M	F	F	F	M	F	M
Apgar score (1, 5 min)	8, 9	8, 9	8, 8	7, 8	9, 9	8, 8	9, 10	6, 7	2, 3	3, 6
Weight for GA	AGA	AGA	AGA	AGA	AGA	SGA	AGA	AGA	SGA	AGA
Mother's disease	No	No	No	No	No	No	No	No	SLE, hypertension	Preeclampsia
Family history	Brother CHD	No	No	No	No	No	No	No	Twin-brother normal	Twin-sister PDA
Coexisting CHD	No	VSD, Ebstein anomaly	PDA	VSD, ASD	No	No	ASD	No	No	No
Ages of onset (d)	2	12	1	6	28	18	11	25	10	7
Symptoms at diagnosis										
Asymptomatic	Yes	No	No	No	No	No	Yes	No	No	No
Heart failure	No	No	No	No	No	Yes	No	No	Yes	Yes
Shock	No	No	No	No	No	No	No	No	No	Yes
Dyspnea	No	No	Yes	Yes	No	Yes	No	No	Yes	Yes
Arrhythmia	No	Yes	No	No	Yes	Yes	No	Yes	Yes	Yes
Others	–	Cyanosis	Cyanosis	–	Cyanosis/cough.	Cyanosis	–	–	–	Cyanosis
Pro-BNP (pg/mL)	135	125.6	32.5	50.3	224.8	531.3	48.3	42.1	168	475.4
CK-MB (ng/mL)	31.2	14.5	8	7.1	24.8	38.8	4.7	3.9	26.1	40.3

GA, gestational age, weeks; M, male; F, female; SGA, small for gestational age; SLE, systemic lupus erythematosus; CHD, congenital heart defect; VSD, ventricular septal defect; ASD, atrial septal defect; PDA, patent ductus arteriosus; pro-BNP, N-terminal pro-Brain natriuretic peptide; CK-MB, creatine kinase-MB; AGA, appropriate for gestational age.

### 3.2 Clinical characteristics

The average age at initial diagnosis was 12 days (range 1–28 days). Clinical manifestations are summarized in Table 1, ranging from asymptomatic to cyanosis, dyspnea, arrhythmia, and heart failure. Two infants (20%) were asymptomatic, five (50%) presented with dyspnea and cyanosis, and six (60%) had arrhythmia (some infants had overlapping symptoms). Heart failure was present in three infants at diagnosis, one with coexisting shock. Routine coagulation screens were within normal limits, and there were no clinically reported episodes of systemic embolism during the follow-up period.

Myocardial injury markers—including pro-Brain natriuretic peptide (pro-BNP) and creatine kinase-MB (CK-MB)—were measured to assess cardiac function (Table 1). The average plasma N-pro-BNP level was 193.3 pg/mL (range 42.1–475.5 pg/mL), with elevated levels (>100 pg/mL) observed in six patients. The average CK-MB level was 19.9 ng/mL (range 3.9–475.4 ng/mL), and it was elevated (>6.6 ng/mL) in eight patients.

ECG characteristics of the patients are summarized in Table 2. ECG abnormalities were present in nine of the 10 infants (90%, except Case 4). The most frequent electrocardiographic abnormalities were complex, including ST-segment depression, T-wave inversions in leads II, III, aVF and V4-V6, premature atrial contractions, and sinus bradycardia. During the clinical course, various arrhythmias were observed in six patients, including incomplete right bundle branch block, paroxysmal supraventricular tachycardia (PSVT), atrial premature beats, atrial tachycardia, and atrioventricular block.

**Table 2:** Electrocardiographic characteristics of neonates With LVNC.

Patient	ECG characteristics	Arrhythmia
1	Neg. T in II, III, aVf, V5-6	No
2	ST dep, CAH	APB
3	ST dep	No
4	Normal	No
5	ST dep, flat T in II, III, aVf, V5-6	Second-degree A-V block
6	Sinus tachycardia, APB	APB
7	Sinus tachycardia, ST dep, RVH	No
8	Neg. T in II, III, aVf, Sinus bradycardia, IRBBB, APB	IRBBB,APB
9	ST dep, PSVT	PSVT
10	Sinus tachycardia, IRBBB	IRBBB

Neg. T, negative T wave; ST dep, ST-segment depression; IRBBB, Incomplete right bundle branch block; PSVT, paroxysmal supraventricular tachycardia; APB, atrial premature beats; A-V block, atrioventricular block; CAH, combined atrial hypertrophy; RVH, right ventricular hypertrophy.

### 3.3 Echocardiographic characteristics

Echocardiographic characteristics are summarized in Table 3. Measurements included cardiac structural and functional diameters. Echocardiograms showed numerous prominent trabeculations and deep intertrabecular recesses communicating with the ventricle in all neonates. Non-compacted myocardial segments were predominantly localized at the ventricular apex, inferior wall, and lateral wall. The average NC/C ratio of ventricular wall at the site of maximal wall thickness was 2.96 (ranging from 2.2 to 4.0). The systolic function of LV, assessed using Simpson's method, was quantified by LVEF and fractional shortening (FS). The average LVEF and FS were 38.5% (range 19%–60%) and 18.8% (range 11%–29%), respectively. The following two-dimensional LV parameters were measured from the parasternal long-axis view: end-diastole (IVSd), left ventricular internal diameter at end-diastole (LVIDd), and left ventricular internal diameter at end-systole (LVIDs). The average LVIDd of 2.26 cm and the average LVIDs of 1.84 cm were both above the normative reference intervals (12), indicating severe LV systolic dysfunction and prominent LV dilatation. The average IVSd was within normative ranges (12). No abnormalities were found in other measures of right ventricular dimensions, such as left atrial diameter. Intracardiac thrombus was not detected in any neonate.

**Table 3:** Echocardiographic measurements in neonates with LVNC.

Patient	AAO	LA	RA	RV	PA	IVS	LVIDd	LVIDs	Site of non-compaction	NC/C ratio	FS (%)	LVEF (%)
	(cm)	(cm)	(cm)	(cm)	(cm)	(cm)	(cm)	(cm)				
1	0.9	1.2	1.8	2	0.6	0.2	1.9	1.6	Apex, IW, LW	2.3	18	40
2	0.9	1.7	1.9	1.9	1.2	0.2	2.3	1.8	All walls	4	21	42
3	0.8	1.5	1.9	1.7	1	0.2	2.1	1.7	IW, LW	2.7	20	40
4	0.9	1.9	2.4	2.2	1.6	0.3	2.5	1.8	IW, LW	2.5	29	60
5	0.9	2.2	1.6	1.7	1.1	0.3	3.0	2.6	Apex, AW, IW, LW	4	14	24
6	0.8	1.4	1.6	1.3	0.6	0.2	2.2	1.8	Apex, IW, LW	2.5	20	42
7	0.8	1.2	2.6	2.2	1.1	0.3	2.2	2.0	Apex, IW, LW	2.2	11	19
8	1.1	1.6	1.6	1.7	1.1	0.3	3.1	2.6	Apex, IW, LW, PW	3	16	37
9	0.8	1.7	1.4	1.5	0.6	0.2	1.8	1.5	Apex, IW, LW, PW	3.3	14	28
10	0.7	1.0	1.1	1.1	0.7	0.2	1.5	1.1	IW, LW, PW	3.1	25	53
Average	0.86	1.54	1.79	1.73	0.96	0.24	2.26	1.84	–	2.96	18.8	38.5

AAO, ascending aorta diameter; LA, left atrial diameter; LV, left ventricle diameter; IVS, intraventricular septum diameter; RA, right atrial diameter; RV, right ventricle diameter; PA, pulmonary artery diameter; LVIDd, left ventricular end-diastolic diameter; LVIDs, left ventricular end-systolic diameter; AW, anterior wall; IW, inferior wall; LW, lateral wall; PW, posterior wall; NC/C ratio, non-compacted/compacted ratio; FS, fractional shortening; LVEF, left ventricle ejection fraction.

### 3.4 Medical treatments during the neonatal period

Treatment options included targeted heart failure and shock during the acute phase. Case 9, who presented with PSVT, received antiarrhythmic drugs. All patients initially received combination therapy, which included intravenous immunoglobulins (IVIG) (2 g/kg), digoxin (5 µg/kg/day), diuretics (spironolactone 1 mg/kg/day), angiotensin-converting enzyme inhibitor (captopril 0.1 mg/kg/day), and L-carnitine (100 mg/kg/day).

### 3.5 Follow-up treatments and short-term outcomes

Two neonates with i-LVNC died during follow-up. Upon analyzing their clinical characteristics, it was noted that both infants presented with heart failure at initial diagnosis, one with coexisting shock. Case 6 died of progressive ventricular dysfunction at 25 days of age. Case 10 presented with poor feeding and slow growth. Over a period of 2 months, his LV function deteriorated, with LVEF declining from 53% to 25%. He died of progressive heart failure at 70 days of age. At the terminal stage, he had severe LV enlargement and dysfunction, with the ventricle assuming a spherical shape.

Eight neonates survived during follow-up lasting up to 1 year. Three ni-LVNC patients (Cases 2, 4, and 7) underwent cardiac surgery at the ages of 26 days, 6 and 10 months, respectively. Following cardiac surgery, non-compaction was no longer present in Case 4 and Case 7. Case 3, who had a PDA, showed spontaneous healing at one of age old. Five infants (Cases 1, 2, 5, 8, and 9) were followed until 1 year of age. During the 6-month treatment period, they were hospitalized monthly to receive IVIG (1 g/kg) and underwent echocardiograms every 3 months. These five infants remained asymptomatic with normal LV systolic function at 1 year of age. In Case 9, a congenital cataract was identified at 4 months, and the ventricular non-compaction had resolved at 6 months of age. At 1 year of age, only Case 2 (with ni-LVNC) showed a decrease in the NC/C ratio (from 4 to 2.5), while the ratio remained unchanged in the other three infants with i-LVNC.

## 4 Discussion

LVNC is a rare, unclassified cardiomyopathy characterized by ventricular walls with excessive trabeculations and deep recesses. This process results from failure of compaction of loose myocardial fibers, producing a sponge-like myocardium with a hypertrophied appearance. It is believed to be a developmental error in the early stages of embryogenesis of the heart, occurring between weeks 5 and 8 of embryonic life (13). Although fetal echocardiography and advanced imaging (including fetal MRI) are critical for detecting major structural anomalies, their sensitivity for identifying isolated, diffuse LVNC—particularly in the absence of associated malformations or overt dysfunction—remains limited (14). Recognition during the neonatal period is challenging due to its highly variable clinical manifestations. This study described neonates diagnosed with LVNC over the past 10 years in an IV neonatal intensive care unit, all of whom were followed for up to 1 year. Our findings demonstrate that early diagnosis and aggressive management are crucial to neonatal LVNC.

In children, LVNC showed no sex predilection (8), while a meta-analysis of 7,600 adults showed a male predominance of 62% (15). Our neonates had a similar male prevalence of 60%. By the original definition of LVNC, the absence of structural heart disease was a prerequisite for diagnosis (16). Recently, studies have shown that LVNC is associated with CHD, ranging from PDA or atrial septal defects/ventricular septal defect to more severe diseases such as Ebstein's anomaly (16, 17). LVNC is classified into isolated and non-isolated forms, with a reported ratio of approximately 6:1 between them (18). The ratio in our cohort was 6:4; the difference was not unexpected since the study involved a small, single-center neonatal population.

LVNC exhibited significant age-related heterogeneity, characterized predominantly by congenital abnormalities in childhood (19). In preterm and SGA neonates, increased trabeculation is frequently a benign, adaptive finding, characterized by preserved systolic function despite geometric or mass alterations (4, 5). In our study, four premature and two SGA infants exhibited systolic dysfunction (LVEF <50%), consistent with pathological LVNC rather than a benign variant. Therefore, the evaluation of systolic function by routine echocardiography is essential to avoid misdiagnosis and guide appropriate management.

In our study, 20% of infants with LVNC had a positive family history of cardiomyopathy, consistent with published rates (16%–44%) (8). Although familial and sporadic forms of non-compaction have been described, cases of LVNC from twins are rarely reported. Notably, two infants in our study were singletons from twin pregnancies: one with a healthy cotwin and the other whose cotwin had a PDA.

Although LVNC is present at birth, it may become clinically overt at any time from infancy through adolescence (20). In our study, the average age for initial diagnosis was 12 days. Patients with LVNC present with a wide spectrum of clinical manifestations, ranging from asymptomatic hypertrabeculation to heart failure as a presentation of infantile cardiomyopathy (2, 13). In our study, two infants were asymptomatic, while others presented with dyspnea, cyanosis, arrhythmia, or heart failure. Consistent with previous reports of rapidly progressive LVNC presenting with heart failure, arrhythmia, or embolic events at initial presentation (21), we observed two fatal neonatal cases. Both presented with heart failure (one with concomitant shock) and died despite immediate aggressive treatment. Mortality was associated with dysfunction, while the structural trait itself was persistent. Heart failure at presentation seemed to show poor prognosis in LVNC patients (2). The absence of thromboembolic events in our neonatal cases may be related to the relatively short-term follow-up and young age of our cohort. Therefore, longer-term monitoring for this complication is crucial.

Elevated plasma levels of myocardial injury markers (proBNP and creatine kinase-MB) were common in our cases. Such markers can be used as valuable supplements to traditional indicators of LV function (22). Consistent with the known myocardial damage effects of neonatal asphyxia, the two preterm infants with severe asphyxia histories exhibited high levels of these markers. This indicates that neonatal asphyxia likely exacerbated myocardial injury in LVNC infants.

ECGs abnormalities were prevalent in 75%–94% of pediatric and adult LVNC patients (23). Consistent with this finding, 90% of neonates in our study exhibited ECG abnormalities, most frequently LV hypertrophy and repolarization abnormality. Ventricular arrhythmias remain a major complication and a leading cause of mortality in patients with LVNC (2, 24). Only one infant in our study presented with PSVT. ECG abnormalities should be considered an adjunctive diagnostic feature of LVNC.

Diagnosis of LVNC in neonates is more difficult than in adults due to its initial variable and non-specific presentation. A previous study demonstrated that younger age at diagnosis, higher immediate LVEDd, non-compact segments, and ECG abnormalities were associated with poor prognosis (9). Therefore, it is critical to perform cardiac imaging and evaluate LV function upon initial presentation.

Echocardiography is usually the method to diagnose LVNC (9). It is essential for clinical echocardiographers to identify suspicious cases as soon as possible. Even in clinically asymptomatic patients, LVNC is associated with impaired systolic function and an increased risk of LV dysfunction (2). Consistently, the infants in our study showed abnormalities related to lower LV systolic function parameters. Notably, even the two asymptomatic neonates showed impaired LVEF in the setting of excessive trabeculation. Two structural parameters of LV (LVIDd and LVIDs) were above the normative reference intervals (12), indicating severe LV systolic dysfunction and prominent LV dilatation in these neonates. The severity of systolic dysfunction was a key predictor of prognosis in LVNC. Fortunately, the surviving infants demonstrated improvement in ventricular systolic function during follow-up.

Considering that the NC/C ratio >2 standard proposed by Jenni et al. (9) had been widely used (7, 25), the ratios of our study met this diagnostic threshold. A previous study suggested that the NC/C ratio might be related to disease severity, indicating high mortality (26). The higher NC/C ratio in our cases did not correlate with severe clinical presentation or death, a conclusion consistent with studies in which the NC/C ratio at diagnosis did not predict outcomes (24, 27). Unlike the reported 16 neonatal cases in which non-compaction was distributed mainly in apical, midseptal, and midlateral segments (7), the non-compacted myocardium in our cohort was predominantly located in the ventricular apex, inferior wall, and lateral wall. Increased NC/C ratios within apical, anterolateral, and inferolateral midpapillary segments have been associated with adverse outcomes (28).

In our study, non-compaction was not found after reversal of structural heart defects in three ni-LVNC infants, but persisted in one case (Case 2). This result is consistent with observations in some ni-LVNC patients (26). Non-compaction in these cases may be attributed to the high-pressure exposure of the ventricle, causing changes to disappear after treatment of the primary disease (26).

Clinical management of LVNC aims to prevent complications such as heart failure, thromboembolic events, life-threatening arrhythmias, and stroke (29). Thus, our acute-phase management focused on symptomatic treatment. This approach is supported by prior studies, including those reporting early cardiac recovery with inotropic support in neonatal NVM (30, 31) and others describing the beneficial effects of the *β*-blocker on LV function, mass, and neurohormonal dysfunction with LVNC (13, 32). Following treatment, left ventricular function improved in five infants (four with i-LVNC and one with ni-LVNC). However, the NC/C ratio was stable in three cases and decreased only in the ni-LVNC infant, consistent with multiple follow-up studies in children showing that the NC/C ratio does not change over time despite therapeutic improvements in cardiac function (7, 26). We also observed complete resolution of non-compaction in the SGA infant. The subsequent non-compaction in this SGA infant was likely a physiological pattern, with the early cardiac dysfunction probably due to other comorbidities in such infants. Mortality in LVNC was associated with cardiac dysfunction rather than the persistent non-compaction morphology itself (24). Our results were consistent with reports that LVNC does not follow an invariably fatal course when diagnosed in the neonatal period (7). Therefore, medical management in neonatal LVNC should prioritize improving cardiac function and preventing complications, rather than reversing the anatomical hallmark of the disease. Early diagnosis and timely medical treatment can lead to favorable remodeling, as evidenced by improvement in ventricular systolic function (29).

Advances in cardiac magnetic resonance (CMR) have improved the ability to diagnose LVNC. However, large-scale studies show that only a small number of patients underwent CMR, despite its potential as an effective diagnostic modality (26). Genetic testing is not routinely performed in clinical practice, even though there is a strong genetic component associated with LVNC, with nearly half of pediatric LVNC patients harboring an identifiable mutation (1, 2). Therefore, in our study, neither CMR nor genetic testing was performed during the 1-year follow-up period.

This study had several important limitations. First, due to the small sample size and retrospective design, the results may be subject to potential biases. Moreover, the small number of non-survivors prevented meaningful statistical comparison with survivors. Second, none of patients underwent CMR or genetic testing during the follow-up period. Third, the lack of consensus regarding optimal disease-modifying treatments for neonates with LVNC limited our ability to systematically evaluate long-term outcomes.

## 5 Conclusions

In conclusion, clinical manifestations of neonatal LVNC are variable, ranging from asymptomatic to dyspnea, cyanosis, arrhythmia, and heart failure. Heart failure at initial diagnosis is associated with poorer prognosis. Physicians and echocardiographers should be familiar with the diagnosis of LVNC to avoid delay. In neonatal LVNC, early and accurate diagnosis and aggressive therapy are crucial for good prognosis, and affected infants require regular follow-up. This study provides valuable information on the clinical characteristics of LVNC and suggests that favorable remodeling can occur after aggressive therapy. Further larger multicenter studies are required to improve diagnostic accuracy and determine the ideal therapeutic approach for this disorder.

## Data Availability

The original contributions presented in the study are included in the article/Supplementary Material, further inquiries can be directed to the corresponding author.
